# Identification of *methyltransferase* and *demethylase* genes and their expression profiling under biotic and abiotic stress in pigeon pea (*Cajanus cajan* [L.] Millspaugh)

**DOI:** 10.3389/fpls.2024.1521758

**Published:** 2025-01-16

**Authors:** Priyanka Kumari, Sougata Bhattacharjee, K. Venkat Raman, Jyotsana Tilgam, Krishnayan Paul, Kameshwaran Senthil, Mahi Baaniya, G. Rama Prashat, Rohini Sreevathsa, Debasis Pattanayak

**Affiliations:** ^1^ National Institute of Plant Biotechnology, Indian Council of Agricultural Research (ICAR), New Delhi, India; ^2^ Division of Molecular Biology and Biotechnology, Indian Agricultural Research Institute, New Delhi, India; ^3^ Division of Genetics and Plant Breeding, Indian Agricultural Research Institute, New Delhi, India

**Keywords:** methylation, demethylation, pigeon pea, m^6^A modification, ALKBHs

## Abstract

The methylation- demethylation dynamics of RNA plays major roles in different biological functions, including stress responses, in plants. m^6^A methylation in RNA is orchestrated by a coordinated function of methyl transferases (writers) and demethylases (Erasers). Genome-wide analysis of genes involved in methylation and demethylation was performed in pigeon pea. Blast search, using Arabidopsis gene sequences, resulted in the identification of two methylation genes (*CcMTA70*, *CcMTB70*), two genes encoding adaptor proteins for methylation (*CcFIPA* and *CcFIPB*) and 10 demethylase (ALKBH) genes (*CcALKBH1A*, *CcALKBH1B*, *CcALKBH1C*, *CcALKBH2*, *CcALKBH8*, *CcALKBH8A*, *CcALKBH8B*, *CcALKBH9*, *CcALKBH10A* and *CcALKBH10B*) in the pigeon pea genome. The identified genes were analyzed through phylogenetic relationship, chromosomal position, gene structure, conserved motif, domain and subcellular location prediction etc. These structural analyses resulted in categorization of MTs and FIPs into one group, i.e.*, CcMTA/B* and *CcFIPA/B*, respectively; and ALKBHs into four groups, viz. *CcALKBH1/*2, *CcALKBH*8, *CcALKBH*9 and *CcALKBH*10. Relative expression analysis of the identified genes in various tissues at different developmental stages revealed the highest level of expression in leaf and the least in root. *CcMT*s and *CcFIP*s had similar patterns of expression, and *CcALKBH10B* demonstrated the highest and *CcALKBH2* the lowest level of expression in all the tissues analyzed. *CcALKBH8* showed the highest induction in expression upon exposure to heat stress, and *CcALKBH10B* demonstrated the highest level of induction in expression during drought, salt and biotic (*Helicoverpa armigera* infestation) stresses. The present study would pave the way for detailed molecular characterization of m^6^A methylation in pigeon pea and its involvement in stress regulation.

## Introduction

Epigenetic modifications on both the DNA and RNAs, without any change of sequence, have emerged as an important player of gene regulation in living organisms (Meyer and Jaffrey, 2014). More than 160 RNA modifications have been identified in mRNA, tRNAs, rRNAs and long non-coding RNA until now (Cantara et al., 2010). Methylation is one of the dominant epigenetic modifications, and modification of adenine through methylation exists as an essential epigenetic mark in both DNAs and RNAs of eukaryotes (Liang et al., 2020). N^6^-methyladenosine (m^6^A), N^1^-methyladenosine (m^1^A), 5-methylcytidine (m^5^C) and 7-methylguanosine (m^7^G) are frequently identified in mRNA (Zhang et al., 2020). m^6^A is the most frequent and reversible modification of RNA (Yue et al., 2019; Duan et al., 2017). It plays a major role in the metabolism of RNA which includes mRNA splicing (Haussmann et al., 2016), control of translation (Luo et al., 2020), stability of RNA (Wang et al., 2014), processing of primary microRNA (Bhat et al., 2020) etc. m^6^A modification occurs mostly at the consensus sequence, RRACH (R = purine and H = A, C, or U), and this is conserved in animals and plants (Bhat et al., 2020). Also, a second consensus sequence, UGUAY (Y = pyrimidine), is found to be conserved only in plants for m^6^A modification. Modulation of RNA methylation takes place with the help of two enzymes, viz. RNA methyl transferases (“writers”) and demethylases (“erasers”). Writers help in installing and eraser in removing the methylation marks (Hu et al., 2019).

There are three broad groups for N^6^A-methyltranferases (Iyer et al., 2016). MT-A70 clade constitutes group 1, consisting of MTA and MTB genes. It is further divided into six different eukaryotic subclades (Iyer et al., 2016). Clades 1–3 are known as METTL3, METTL4 and METTL14, respectively and these three clades are conserved in higher eukaryotes. Clades 4–6 of group 1 occur in unicellular photosynthetic eukaryotes, basal fungi, and haptophyte algae (Iyer et al., 2016). The other two groups, group 2 and group 3, show independent transfer from bacteria and have restricted distribution (Iyer et al., 2016). Group 2 has been found in archaeal dsDNA viruses and mycobacterium, which are often seen, fused to RNA-binding PPR domains. Group 3 has been observed only in case of the heterolobosean Naegleria (Iyer et al., 2016). m^6^A modification is carried out by a core heterodimer formed by METTL3 and METTL14, whereas METTL4 is a DNA methylase (Greer et al., 2015). N^6^A methylation is facilitated by an adaptor protein, WTAP (Wilm’s tumor-associated protein), which stabilizes the heterodimer (formed by METTL3 and METTL14) in the nuclear speckle (Ping et al., 2014) and several co-factors like KIAA1429 and an RNA binding motif protein, RBM15/RBM15B. WTAP is found in the animal system, and its ortholog, FIP [FK506-binding protein 12 (FKBP12) interacting protein (FIP), FIP37 in case of Arabidopsis], is present in plants (Shen et al., 2016).

Demethylase (eraser) has been studied extensively in animals, but it is yet to be characterized in detail in plants. Nine demethylases have been reported in humans. Eight demethylases belong to ALKBH family (ALKBH 1-8) and the other one is FTO (Fat mass and obesity associated). Due to selectivity towards the substrate, functional diversity arises among the demethylases (Marcinkowski et al., 2020). Phylogenetic analysis could not detect presence of any FTO ortholog in plant system, but many orthologs of *ALKBH5* are identified in Arabidopsis (Liang et al., 2020).

AlkB homologs (ALKBH) are specific demethylases, which are members of the dioxygenase superfamily and require Fe^2+^and α-ketoglutarate for demethylation catalysis of various substrates, viz. proteins, mRNA, tRNA, snRNA, ds/ss DNA, etc (Trewick et al., 2002). AlkB protein was initially found in *Escherichia. coli* (*Ec*AlkB) (Kataoka et al., 1983). This protein has de-alkylation activity that repairs the damage caused by alkylating agents. Repairing of 3-methylcytosine (3-meC) and 1-methyladenine (1-meA) base modifications is more efficient compared to that of 1-methylguanine (1-meG) and 3-methylthymine (3-meT), which undergo less efficient repairing process. A single gene encoding ALKB is present in *E. coli*, but many *ALKBH* gene families are present in animals and plants (Marcinkowski et al., 2020). Also, ALKBH has repairing as well as regulatory roles in eukaryotes indicating a broader range of functions.

Fourteen ALKBH families have been identified in Arabidopsis using bioinformatic tools (Mielecki et al., 2012; Ougland et al., 2015; Marcinkowski et al., 2020). These Arabidopsis ALKBH proteins have functional diversity and act on different substrates. ALKBH1D is present in chloroplast. ALKBH2 does the repairing of 1-meA and 3-meC, ALKBH8 takes part in modification of tRNA by hydroxylating mcm5U to (S)-mchm5U, ALKBH9A and ALKBH10A are related to abiotic stresses and ALKBH9B and ALKBH10B have N^6^ demethylation activity (Eraser).

Methylase –demethylase system in Arabidopsis is involved in regulation of stem cell fate determination (Shen et al., 2016); embryo development (Zhong et al., 2008); and trichomes and leaf morphology (Wei et al., 2018). m^6^A modification is found to affect sporogenesis in rice (Zhang et al., 2019). However, the function of the methylase-demethylase system is yet to be studied in many agriculturally important crops.

Pigeonpea (*Cajanus cajan*) is a climate resilient orphan crop with rich source of proteins, essential amino acids and vital minerals. It is an important pulse crop grown in tropical and subtropical areas. India is the largest producer of pigeon pea in the world and presently it is grown on over 5.65 m ha in India (FAOSTAT, 2022). Despite the development of high yielding varieties through breeding efforts the productivity is stagnant at around 825kg/ha (FAOSTAT, 2022) which is not sufficient to meet the demand of ever increasing human population. The methylase-demethylase system has not been studied in pigeon pea. It has been reported that m^6^A modification has a role in abiotic and biotic stresses (Miao et al., 2020). Even though, pigeon pea considered as resilience to abiotic stresses, many factors like moisture and water logging stress affects the crop physiology and productivity. In North Western part of India, pigeon pea crop suffers from salinity stress (Choudhary et al., 2011). Extreme drought and heat conditions particularly at semi-arid areas, during the seedling and reproductive stages in pigeon pea plants often leads to yield loss (Bakala et al., 2024). Among Biotic stresses, infestation of pod borer (*H armigera* Hubner) poses major challenge to the pigeon pea productivity. With the availability of genomic sequence and annotations, there is a potential way to explore the genes to enhance the tolerance using advanced genomic, genome editing and speed breeding tools. So, considering the diverse role of methylase-demethylase system we made an attempt to identify the methyl transferase and demethylase in pigeon pea and a bioinformatics analysis was conducted for identification and analysis of methylase and demethylase genes in pigeon pea. The expression pattern of the identified genes was analyzed in different tissues and upon exposure to biotic and abiotic stress conditions.

## Materials and methods

### Identification of *CcMTs*, *CcFIPs* and *CcALKBHs* and retrieving sequence from database

Arabidopsis MT, FIP and ALKBH cDNA and protein sequences were downloaded from the ensemble (https://plants.ensembl.org/index.html) using the gene ID provided in the literature (Ougland et al., 2015). The protein sequence was then used for blast search in the Legume Information Database (https://www.legumeinfo.org/) to see the orthologous proteins in pigeon pea. E-value threshold was kept at zero for blast search with 98-100% coverage.

### Determination of protein weight and other parameters using Expasy

The different Expasy (https://www.expasy.org/) tools like ProtParam, compute pI/Mw etc was used to have a basic understanding of the identified genes in terms amino acid length, molecular weight, iso-electric point, GRAVY (Kyte and Doolittle, 1982), instability index and aliphatic index.

### Determination of chromosomal location

The chromosomal position was identified from the LIS database (https://www.legumeinfo.org/) and subsequently, the locus ID was also identified from the NCBI database (https://www.ncbi.nlm.nih.gov/). Chromosome map was constructed using MapGene2Chrom web v2 (http://mg2c.iask.in/mg2c%5Fv2.1/).

### Construction of phylogenetic tree for MTs, FIPs and ALKBHs of pigeon pea

A phylogenetic tree of the identified proteins was constructed to see their relative closeness. MEGA11 software was used for the phylogenetic tree construction (Tamura et al., 2021). First of all, after identification and retrieval of all the sequences Clustal omega (https://www.ebi.ac.uk/Tools/msa/clustalo/) was used to check for similarity among sequences. Further of full-length amino acid sequences of *Arabidopsis thaliana, Oryza sativa, Glycine max and C. cajan* were fed to MEGA and there again multiple sequence alignment was performed with ClustalW tool. The IDs for Oryza sativa and glycine max is provided in **Table 1**.While aligning the sequences of four species of crops in MEGA, alignments were made selecting “with gap option” and during construction of the phylogenetic tree, gap parameters were selected as ‘Use all site’. Phylogenetic tree was constructed using the Maximum Likelihood method and JTT matrix-based model taking bootstrap value 1000 (Tamura et al., 2021). For visualization of the phylogenetic tree an ‘Interactive Tree Of Life’ (iTOL) v6 (https://itol.embl.de/) was used.

**Table 1:** List of gene ID of methyltransferase and demethylase genes for rice and soybean.

Gene Type	Name	*Oryza sativa* gene ID	*Glycine max* gene ID
WRITER	*MTA*	LOC_Os02g45110	Glyma.16G033100
Methyltransferase	*MTB*	LOC_Os01g16180	Glyma.20G161800
	*FIP37*	LOC_Os06g27970	Glyma.17G086600
ERASER	*ALKBH1A*	LOC_Os03g60190	Glyma.18G006200
Demethylase	*ALKBH1B*	LOC_Os11g29690	Glyma.19G263000
	*ALKBH1C*		
	*ALKBH1D*		Glyma.01G129600
	*ALKBH2*	LOC_Os06g17830	Glyma.09G014800
	*ALKBH6*	LOC_Os10g28410	Glyma.09G156400
	*ALKBH8*	LOC_Os04g51360	Glyma.04G107300
	*ALKBH8A*	LOC_Os11g43610	Glyma.09G217100
	*ALKBH8B*		Glyma.14G026500
	*ALKBH9A*	LOC_ Os06g04660	Glyma.17G220300
	*ALKBH9B*		
	*ALKBH9C*		Glyma.14G106000
	*ALKBH10A*	LOC_Os05g33310	Glyma.02G149900
	*ALKBH10B*	LOC_Os10g02760	

### Identifying gene structure and conserved motif

For identification of gene structure GSDS 2.0 was used (http://gsds.gao-lab.org/). This gave the idea of exon-intron structure in *MTs*, *FIPs* and *ALKBHs* genes. The conserved motifs of the protein were examined using the MEME online software tool (https://meme-suite.org/meme/). The motif number was kept as 20. The motif width range was kept as minimum 6 and maximum of 50 (6-50) and in site distribution zero or one occurrence per sequence was selected.

### Prediction of conserved domain and sub-cellular localization of MTs, FIPs and ALKBHs of pigeon pea

The conserved domain of the genes was predicted using an online ‘CD Search tool’ (https://www.ncbi.nlm.nih.gov/Structure/bwrpsb/bwrpsb.cgi). The sub-cellular localization of pigeon pea MTs, FIPs, and ALKBHs was predicted using the WoLF PSORT web tool (https://wolfpsort.hgc.jp/).

### Identification of cis-elements in the promoter region of *MT*s, *FIP*s and *ALKBH*s and prediction of their methylation position

Upstream genomic sequences of 2 kb from transcription start site (including 5’ UTR) were retrieved from the LIS database for *MT*s, *FPI*s and *ALKBH*s. Cis-regulatory elements were identified using the Plant Pan v3.0 (http://PlantPAN.itps.ncku.edu.tw/). Data obtained from the web tool was analyzed in MS Excel V.2013 and visualized in the TB tool (https://bio.tools/tbtools). Upstream sequences for *MT*s, *FPI*s and *ALKBH*s were analyzed to predict the m^6^A- methylation using the EpiSemble R-package v.0.1.1 (http://cabgrid.res.in:5799/). MethSemble 6mA tool was used in EpiSemble R-package to predict the methylation site. This package uses three models viz gradient boosting, random forest and Support vector machine.

### Biotic and abiotic stress conditions

Pigeon pea genotype, Pusa 992, was grown in the net house in a 4-inch pot (loamy soil) under natural day length (14hr light and 10 hr. dark) and temperature(30-32 °C) in July 2023 at ICAR-NIPB, New Delhi. Initially watering was done on every alternate day up to three leaved stage. After that when soil used to dry based on that watering was done. Plants were grown in triplicates. For heat stress, 20 days old seedlings are kept in a heat chamber at 42 °C and 60% relative humidity. Plants were kept for 6 hrs. (from 11:00 am to 5:00 pm) for two days (**Supplementary Figure 1**). After the second day leaf samples were taken. For drought stress, 20% PEG 6000 was prepared by adding 200gm of PEG 6000 in 1000ml of autoclaved water and 100ml of PEG was given per pot which contained a single plant. (**Supplementary Figure 1**). For salt stress 150 mM of NaCl was prepared by adding 8.766 gm in 1000 ml autoclaved water. and 100 ml of the solution was given to 20 days old seedlings (single plant in one pot) (Mi et al., 2024; Dokka et al., 2024) (**Supplementary Figure 1** **).** *Helicoverpa armigera* was used for biotic stress. Larvae were obtained from an in-house culture facility The larvae of *H. armigera* larvae were raised on an artificial diet with a 16 h light and 8 h dark photoperiod, at a temperature of 26± 1°C and 70-80% relative humidity was maintained. The second instar larvae of the polyphagous insect pest, *Helicoverpa armigera*, was reared on leaves of 20 days old seedlings of pigeon pea. Larvae were given 7 hrs. starvation and then two larvae were released per pot (1 Plant in each pot). Plants were covered with the perforated polythene which was secured with rubber band on the pots to prevent escape of larvae. After four days of infestation leaf samples were collected.

### Plant material and qPCR analysis of identified genes in tissue-specific manner and under biotic and abiotic stress conditions

For tissue-specific expression studies different tissues were collected at different stages, but for stress related studies leaf tissues were collected from 20 days old seedlings. For abiotic stress-induced plants leaf samples were collected after 48 hours of treatment. Leaf samples were collected after 4 days of *H. armigera* infestation for gene expression study under biotic stress. Total RNA was isolated from different tissues (leaf, roots, internode, shoot apical meristem, flower apical meristem and immature pod) for tissue-specific qPCR and from leaf samples for stress-specific qPCR using RNA isolation kit (Genes2Me; India) according to the ‘manufacturer’s instruction. Isolated RNA was treated with DNaseI (RNase free) to remove any genomic DNA contamination. The quality of total RNA was checked using a Nanodrop spectrophotometer (Thermo Scientific**).** Total RNA was then immediately stored at -80°C. cDNA was prepared using a PrimesScript cDNA synthesis kit (TaKaRa) and stored at -20 °C for further use. *MT*s, *FIP*s and *ALKBH*s-specific primers (**Supplementary Table 2**) were designed using the IDT web tool (https://www.idtdna.com/). qPCR assay was performed in Light Cycler 96 PCR detection system (Roche, Basel, Switzerland) using TB green master mix (TaKaRa) using the following conditions: initial denaturation at 95 °C for 5 min, 40 cycles of amplification, each cycle of 95 °C for 30 sec, 60 °C for 30 sec and 72 °C for 20 sec. Also, three biological and three technical (cDNA replicates) replicates were taken for each sample. The *CcIF4* was used as a reference gene (Bhattacharjee et al., 2023). The Sequence of the internal primer pair for the reference gene is included in the **Supplementary File** (**Supplementary Table 2**). The relative abundance of *CcMTs, CcFIPs* and *CcALKBHs* was calculated using the 2^–ΔΔCt^ method (Livak and Schmittgen, 2001).

### Statistical analysis

For the gene expression study, three biological (separate plants grown in separate pots under different abiotic and biotic stress) and three technical replicates were taken. Wherein, an equal amount of each biological replicate was pooled for RNA isolation. Mean values were given with an error bar (standard error of means) for all the parameters. At 5%, the least significant difference (LSD) was calculated to see the significance of different treatment effects. After that, the significance level between and among the treatments in each experiment was checked by performing a range test.

## Results

### Identification of Arabidopsis MT, FIP and ALKBH orthologs of pigeon pea

The sequence information of methyl transferase and demethylase was retrieved from the LIS database and cross-checked through blast at NCBI database, which gave the Locus ID of respective genes. Arabidopsis has 2 *MT*, 1 *FIP* and 14 *ALKBH* genes, whereas in pigeon pea, 2 *MT*, 2 *FIP* and 10 *ALKBH* genes were identified (**Table 2**).

**Table 2:** List of methyl transferase and demethylase genes involved in RNA methylation in pigeon pea.

Gene Type	Name	Arabidopsis gene ID	Target RNA	Function	Pigeonpea ortholog	Animal homolog
WRITER Methyltransferase	*MTA* *MTB* *FIP37*	At4g10760 At4g09980 At3g54170			Cc_02310 Cc_04693 Cc_26978	*METTL3* *METTL14* *WTAP*
ERASER Demethylase	*ALKBH1A* *ALKBH1B* *ALKBH1C* *ALKBH1D* *ALKBH2* *ALKBH6* *ALKBH8* *ALKBH8A* *ALKBH8B* *ALKBH9A* *ALKBH9B* *ALKBH9C* *ALKBH10A* *ALKBH10B*	At1g11780 At3g14140 At3g14160 At5g01780 At2g22260 At4g20350 At1g36310 At1g31600 At4g02485 At1g48980 At2g17970 At4g36090 At2g48080 At4g02940	tRNA mcm5U tRNA mcm5U m6A m6A	Viral infection Flowering	Cc_00082 Cc_01989 Cc_08628 Cc_06617 Cc_06586 Cc_13896 Cc_12521 Cc_11071 Cc_03631 CC_07468	*ALKBH1* *ALKBH2* *ALKBH6* *ALKBH8* *ALKBH5*

### Determination of protein weight and other parameters using the Expasy database

This exercise provided a complete framework of basic information on iso-electric point (pI), molecular weight (MW), instability index (II), aliphatic index (AI) and grand average of hydropathicity (GRAVY) of the proteins identified in pigeon pea (**Table 3**). When the protein sequence of identified MTs, FIPs and ALKBHs of pigeon pea was analyzed it was found that there was variation in the genes. For instance, in case of predicted protein length, the amino acid sequence varies from 761 aa to 1089 aa for MTs, 337 aa to 338 for FIPs and 205 aa to 515 aa for ALKBHs family of pigeon pea. The iso-electric point for MTs ranged from 6 to 7, and for FIPs, it was between 5 and 6. The iso-electric point ranged from 5.57 to 8.7 for ALKBHs with CcALKBH1C having the highest PI and *Cc*ALKBH8B the lowest. All proteins of MTA, FIPs and ALKBH were hydrophilic as confirmed by GRAVY. Also, instability index analysis showed that MTA was more stable than MTB, and FIPA was more stable than FIPB. Among ALKBH proteins *CcALKBH8* was the most stable, and *CcALKBH8B* was the least stable protein. Aliphatic index analysis indicated that MTA and FIPA had more aliphatic amino acids compared to MTB and FIPB, respectively. *CcALKBH8* had the highest aliphatic index and *CcALKBH9* had the lowest aliphatic index. The higher aliphatic index, the better the thermo-stability of protein.

**Table 3:** Details of identified proteins involved in methylation-demethylation in pigeon pea.

Gene name	Protein length	Molecular Weight (Kd)	PI	GRAVY	II	AI
*CcMTA*	761	84.24	6.08	-0.458	49.48	76.49
*CcMTB*	1089	120.9	6.87	-1.185	53.52	42.65
*CcFIPA*	337	38.37	5.64	-0.878	52.01	71.31
*CcFIPB*	338	38.38	5.17	-0.891	60.75	69.02
*CcALKHB1A*	345	38.93	8.48	-0.448	50.16	81.68
*CcALKHB1B*	481	53.42	8.24	-0.587	53.26	69.46
*CcALKHB1C*	276	30.51	8.64	-0.550	59.11	73.44
*CcALKHB2*	205	22.28	5.80	-0.640	58.79	71.37
*CcALKHB8*	386	42.6	6.18	-0.165	31.87	88.65
*CcALKHB8A*	342	38.28	6.92	-0.314	54.79	84.39
*CcALKHB8B*	219	25.11	5.50	-0.376	70.84	85.53
*CcALKHB9*	480	53.5	6.18	-0.673	45.13	68.02
*CcALKHB10A*	511	56.32	6.09	-0.370	52.72	75.42
*CcALKHB10B*	515	56.71	5.71	-0.348	51.44	76.95

### Determination of chromosomal location using LIS database and construction of chromosomal map using MapGene2Chrom web v2.1

From the analysis of the chromosomal position of the genes encoding pigeon pea MTs, FIPs and ALKBHs it was found that all the genes were localized within six chromosomes, viz. chr.01, chr.02, chr.03, chr.05, chr.06 and chr. 11. However, the majority of the genes were localized on the chr.03 (**Table 4**).

**Table 4:** Specific chromosomal location of the identified methylase and demethylase genes in pigeon pea.

Gene name	Locus ID	Start position	End position	Location
*CcMTA*	LOC109796915	17132963	17136525	Chr1
*CcMTB*	LOC109806648	29180464	29187590	Chr2
*CcFIPA*	LOC109807646	722001	728778	Chr3
*CcFIPB*	LOC109796750	12049020	12058710	Chr11
*Cc ALKBH 1A*	LOC109795359	1402570	1404882	Chr1
*Cc ALKBH 1B*	LOC109816189	73711	75566	Chr1
*Cc ALKBH 1C*	LOC109798028	29340539	29341174	Chr3
*Cc ALKBH 2*	LOC109800658	1113721	1116509	Chr3
*Cc ALKBH 8*	LOC109796724	1470740	1472313	Chr3
*Cc ALKBH 8A*	LOC109815811	95041	98599	Chr6
*Cc ALKBH 8B*	LOC109795028	14318278	14318937	Chr5
*Cc ALKBH 9A*	LOC109818090	212627	216263	Chr5
*Cc ALKBH 10A*	LOC109795864	24839450	24844871	Chr2
*Cc ALKBH 10B*	LOC109795704	16277867	16284890	Chr3

**Figure 1:**
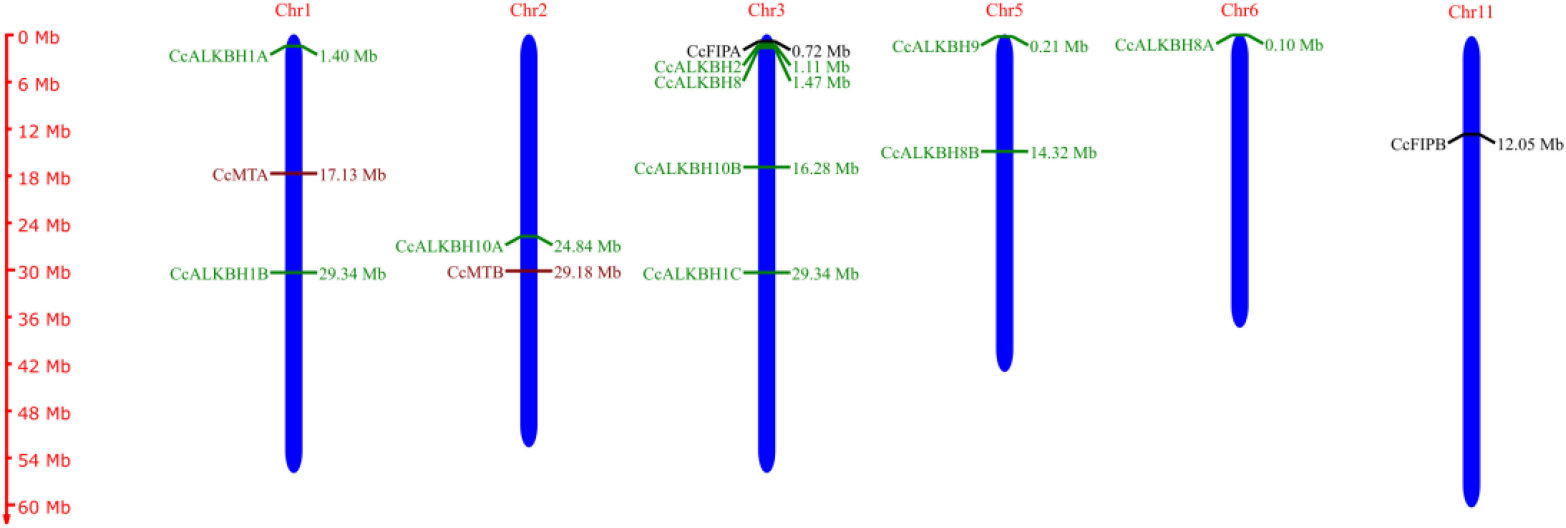
Chromosomal map (made using Map Gene 2 chromosome software) depicting chromosomal locations of the identified genes involved in methylation-demethylation in pigeon pea. The maroon colour indicates *CcMTs*, green indicates *CcALKBH*s and black indicates *CcFIPs*.

A chromosomal map had been constructed showing the distribution of genes on chromosomes. Cc*MTA* and Cc*MTB* were located on chromosomes 01 and 02, respectively. Cc*FIPA* and Cc*FIPB* were located on chr.03 and 11, respectively. Two ALKBH genes, *CcALKBH1A* and *CcALKBH1B*, were located on chr.01, one ALKBH gene, *CcALKBH10A*, was on chr.02, and four genes, viz, *CcALKBH1C, CcALKBH2, CcALKBH8* and *CcALKBH10B*, were onchr.03. *CcALKBH8B, and CcALKBH9* were present on chr.05 and *CcALKBH8A* was found on chr. 06 (**Figure 1**).

### Phylogenetic analysis of identified genes in pigeon pea

**Figure 2:**
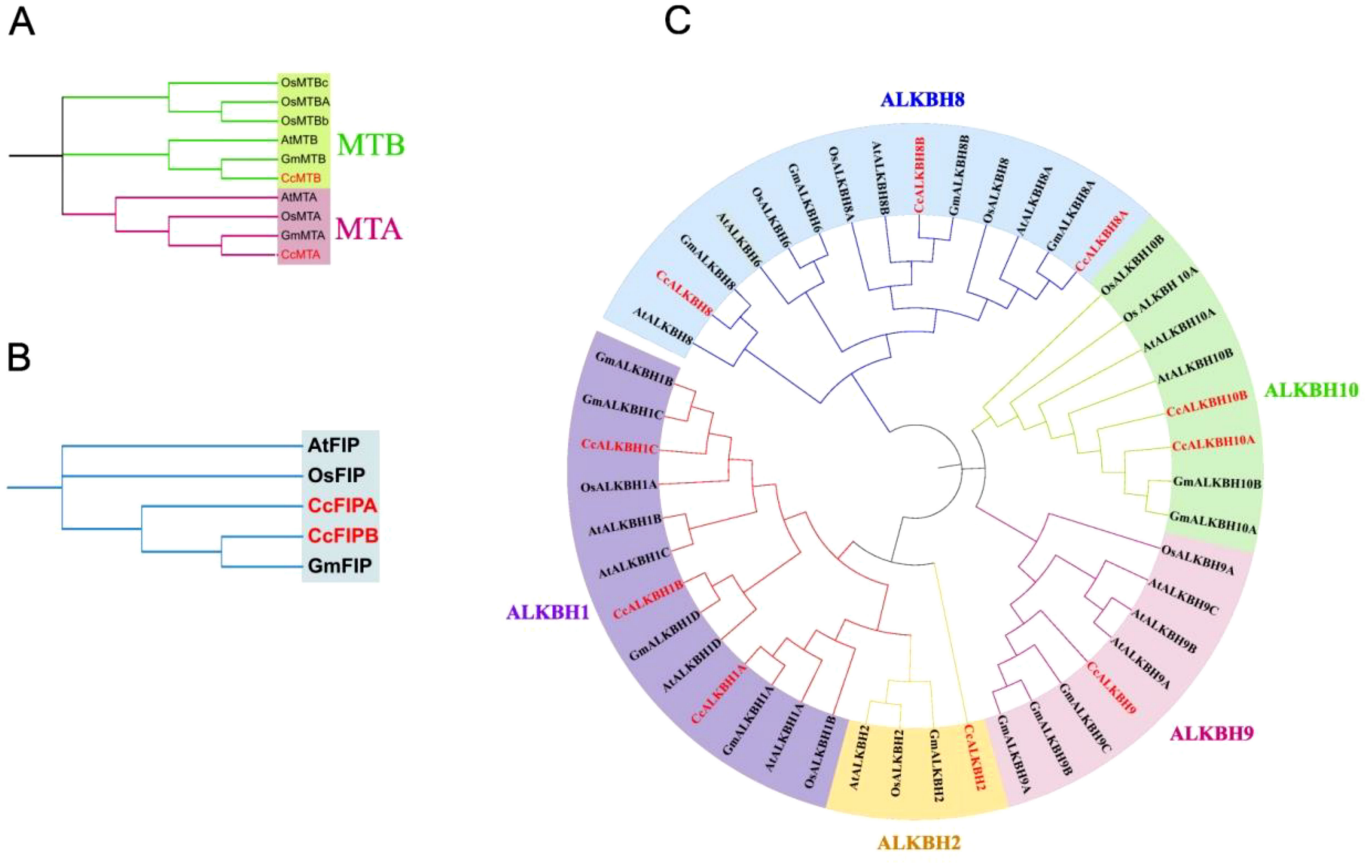
Phylogenetic tree showing the relationship and closeness among the identified methylation-demethylation genes of *Arabiopsis thaliana*, rice (*Oryza sativa*), soybean (*Glycine max*) and pigeon pea (*Cajanus cajan*). **(A)** *MT*s, **(B)** *FIP*s, **(C)** *ALKBH*s. Phylogenetic tree was constructed using MEGA11.0 software by selecting the Maximum likelihood and keeping the bootstrap value at 1000.

Deduced protein sequences of MTs, FIPs and ALKBHs from Arabidopsis (*A. thaliana*), rice (*O. sativa*), soybean *(G. max*) and pigeon pea (*C. cajan*) were taken and a phylogenetic tree was constructed using MEGA11 to find out the relationships between the identified genes and to see the evolutionary relics (Tamura et al., 2021) (**Figure 2** **).** The tree sub-clades were clubbed into groups to understand their evolutionary relations. One group for MTs and FIPs, viz. MTA/B and FIPA/B, respectively, and four groups for ALKBHs were made, viz, (ALKBH1/2, ALKBH8, ALKBH9, ALKBH10) following earlier nomenclature (Marcinkowski et al., 2020). The number of genes of MTs and FIPs was almost the same in above mentioned species. However, the number of *ALKBH* genes varied among species. The highest number of *ALKBH* genes (14) was found in Arabidopsis, while the least number of ALKBHs (10) was found in pigeon peas. The ALKBH6 group was found absent in pigeon pea. The ALKBH1 group had the highest number of genes (4), while ALKBH2 and AlKBH9 had the lowest number of genes (1). ALKBH10 was similar and related to m^6^A RNA demethylation in Arabidopsis.

### Identification of gene structure and conserved motifs for *MT*s, *FIP*s and *ALKBH*s

**Figure 3:**
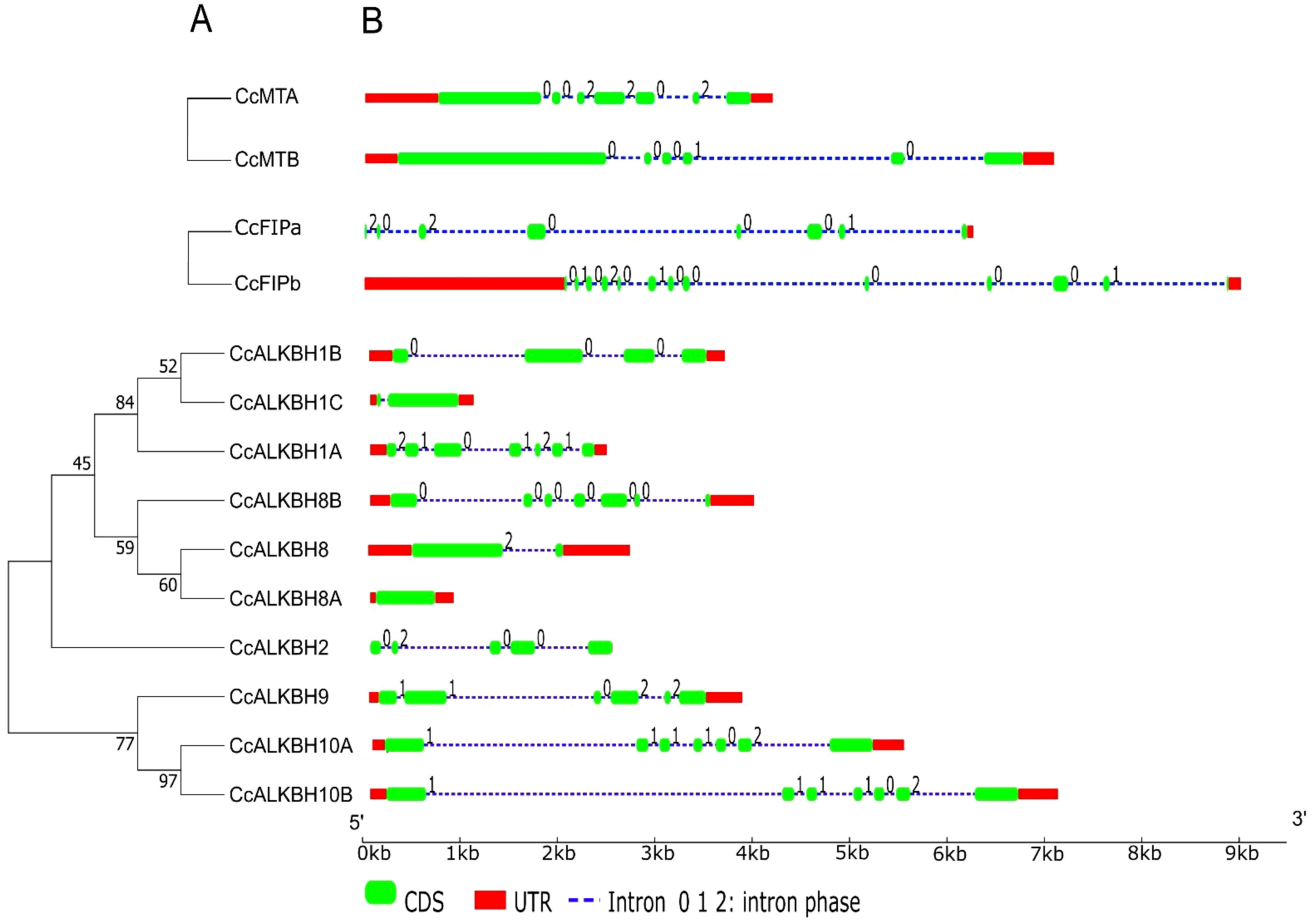
Phylogenetic tree showing gene structure of the identified genes of pigeon pea involved in methylation-demethylation. **(A)** Phylogenetic tree of identified pigeon pea MTs, FIPs and ALKBHs proteins. **(B)** Gene structures of *CcMTs, CcFIPs and CcALKBHs*. The exons, introns and UTRs were represented by red rectangles, black lines and green rectangles respectively. 0, 1 and 2 represent the intron phase of the respective genes.

Gene structure plays a major role in the evolution of gene families. A phylogenetic tree was constructed using the neighbor joining method grouped *CcALKBs* into 4 paralogous clades. The members of *Cc*ALKBH1, *Cc*ALKBH2 and *Cc*ALKBH8 were 3 distinct clades whereas, the members of *Cc*ALKBH9 and *Cc*ALKBH10 together grouped as a separate clade (**Figure 3A**). Analysis of the exon-intron structure of *MT*s revealed that seven and six exons were present in *MTA* and *MTB*, respectively, but intronic portion was more in *MTB* (**Figure 3B**). In case of *FIPA* and *FIPB* there were eight and thirteen exons, respectively, and for *FIPA*, UTR was found only at the 3’ end (**Figure 3B**). Intronic portion was found to be more in *FIPB*. Further, for ten *ALKBH*s, it has been found that variations were present among the genes. Seven exons were present in five of the *ALKBH*s, viz. *CcALKBH1A*, *CcALKBH8A*, *CcALKBH9*, *CcALKBH10A* and *CcALKBH10B*; and the rest five *ALKBH*s, viz. *CcALKBH2*, *CcALKBH1B*, *CcALKBH1C*, *CcALKBH8* and *CcALKBH8B*, had varying numbers of exons (five to one, respectively). *CcALKBH10A* and *CcALKBH10B* contained the largest intronic regions. It was noticed that the *CcALKBH2* gene was devoid of any UTR region (**Figure 3B** **).**

**Figure 4:**
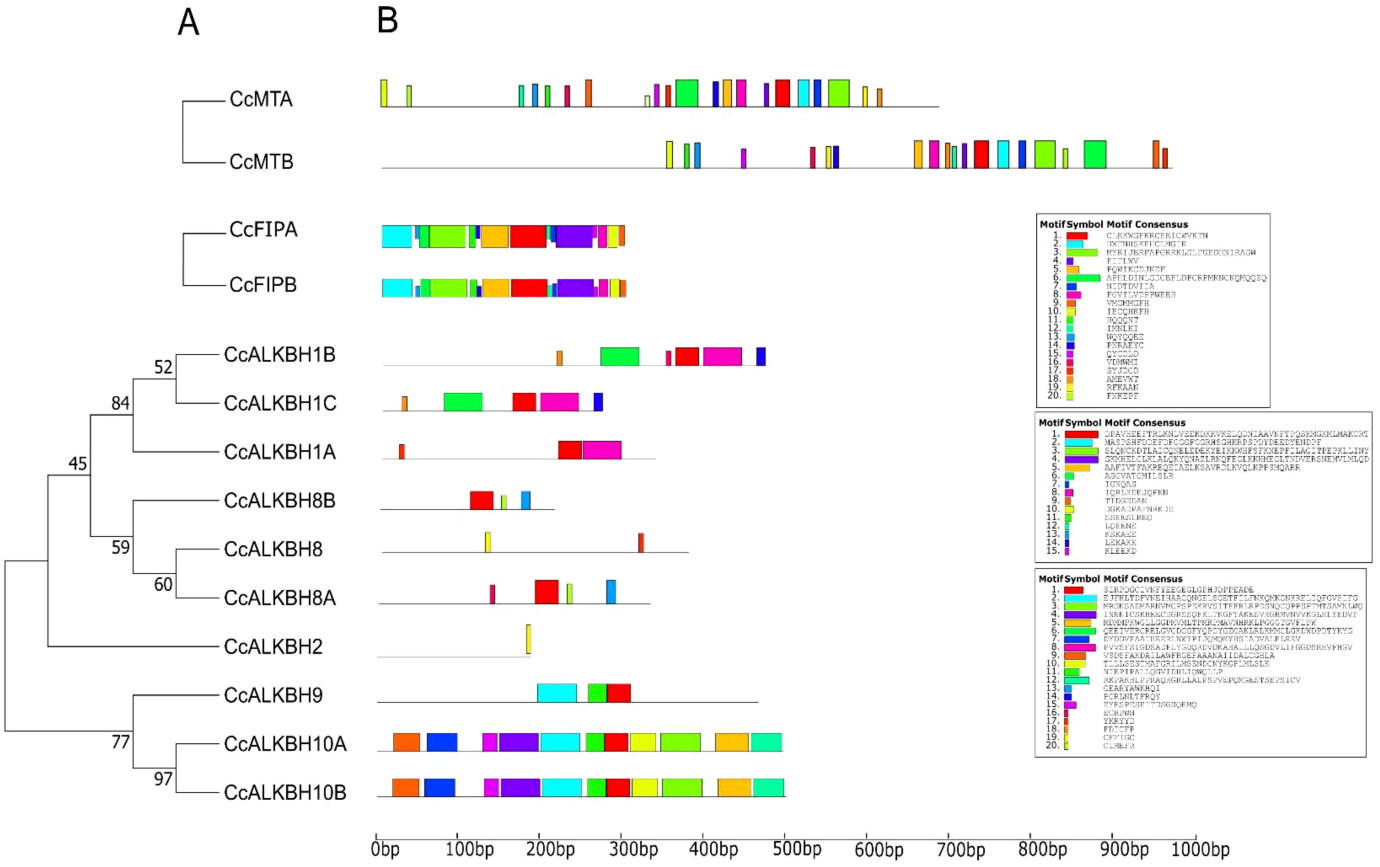
Phylogenetic tree showing conserved motifs. **(A)** Conserved motifs are present in the identified MTs, FIPs and ALKBHs proteins of pigeon pea. **(B)** Distribution of identified conserved motifs of *CcMTs, CcFIPs, and CcALKBHs* genes of pigeon pea. Different motifs are shown in different colors.

The MEME suite was used for conserved motif analysis, and 20 motifs were identified. According to the phylogenetic analysis (**Figure 4A** **),** motif distribution was found conserved for closely related genes as shown in **Figure 4B**. All 20 motifs were found to be present in both the *MT*s, but their distance varied. *FIP*s had all the motifs conserved and at the same distance. For *ALKBH*s, *CcALKBH10A* and *CcALKBH10B* had the greatest number of genes conserved at the same distance (**Figure 4B** **).** Motif 1 was found to be conserved in all the genes except for *CcALKBH2* and *CcALKBH8*. Motif 20 was specific to *CcALKBH8A* and *CcALKBH8B*. Similarly, motif 18 was specific to *CcALKBH1B* and *CcALKBH1C*, and motif 19 was specific to *CcALKBH2* and *CcALKBH8* (**Figure 4B** **).** *CcALKBH2* and *CcALKBH8* had shown the least conservation of motifs which might be an indication of performing different functions.

### Domain and subcellular localization prediction

**Figure 5:**
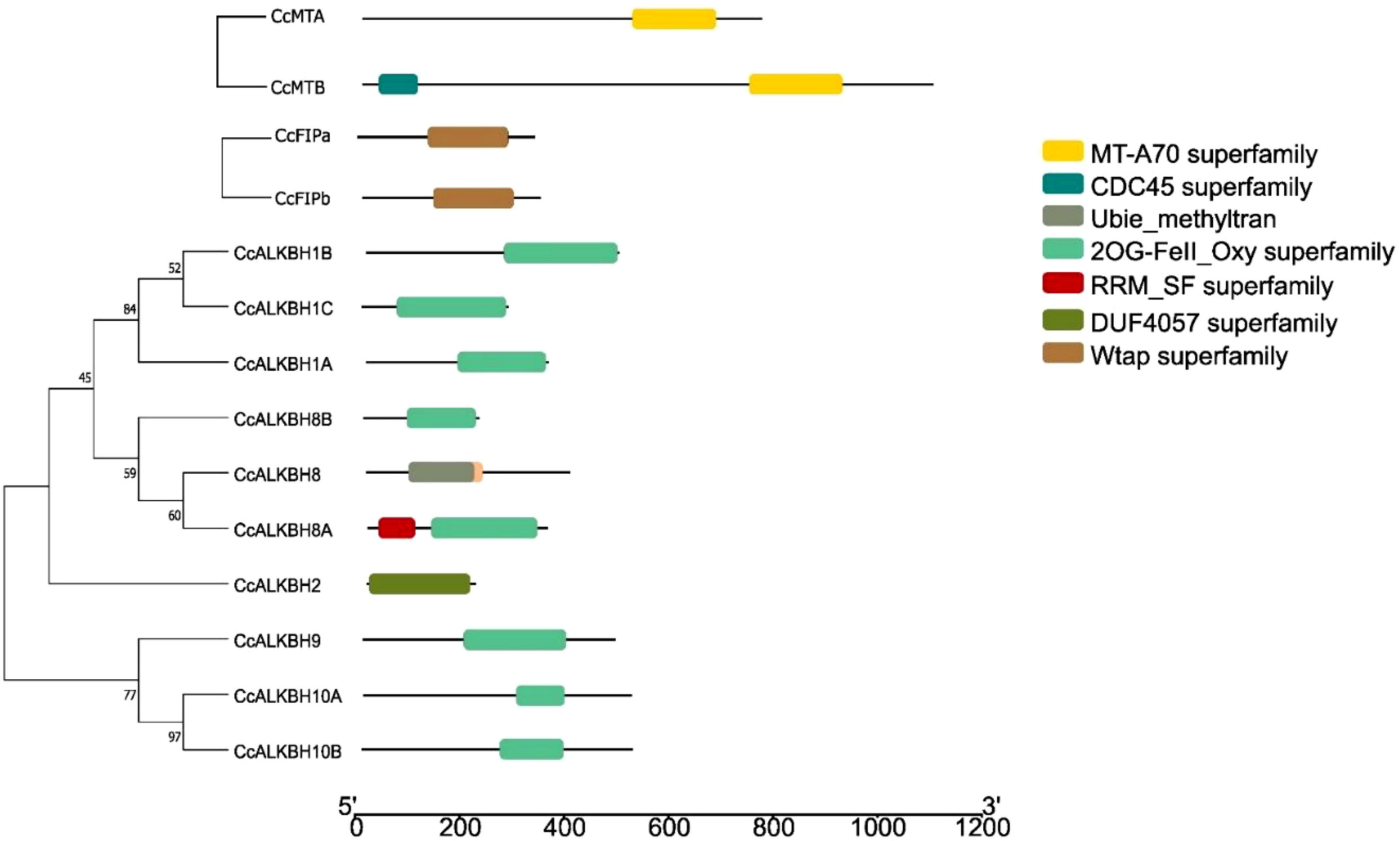
Phylogenetic tree constructed based on conserved domains of identified *CcMTs, CcFIPs* and *CcALKBHs* genes of pigeon pea. Different domains are shown in different colors.

Domains are the self-stabilizing polypeptide chain that works independently. It was observed that MTs (*CcMTA* and *CcMTB*) contained domain MTA-70 which is a major domain involved in methylation (**Figure 5**). Microbial surface components recognizing adhesive matrix molecules domain was found in FIPs, *viz. CcFIPA* and *CcFIPB* (**Figure 5**). Most of the ALKBHs contained 2OG-FeII_Oxy_2 domain which is a characteristic feature of the *ALKBH* gene family. Although the 2OG-FeII_Oxy_2 domain was absent in *CcALKBH2*, it had a completely different domain of DUF4057 superfamily, which is yet to be characterized (**Figure 5**). Apart from this, *CcALKBH8A* was found to have an RRM (RNA recognition motif) domain, which is an essential domain involved in tRNA modification. In addition, *CcALKBH8* also had a methyl transferase domain. So, it might have a role in both demethylation and methylation.

Regarding subcellular location, it was found that MTs, FIPs and a majority of ALKBHs had nuclear localization signal. However, a few ALKBHs, viz*. CcALKBH1B, and CcALKBH10B*, had chloroplast targeting signals, *CcALKBH8* had signal peptide sequence targeting the plasma membrane (**Supplementary Table 1**), and *CcALKBH9* had both nuclear and cytoplasm localization signals.

### Identification of cis-regulatory elements and m6A-methylation sites in the promoter region of *MT*s, *FIP*s and *ALKBH*s

**Figure 6:**
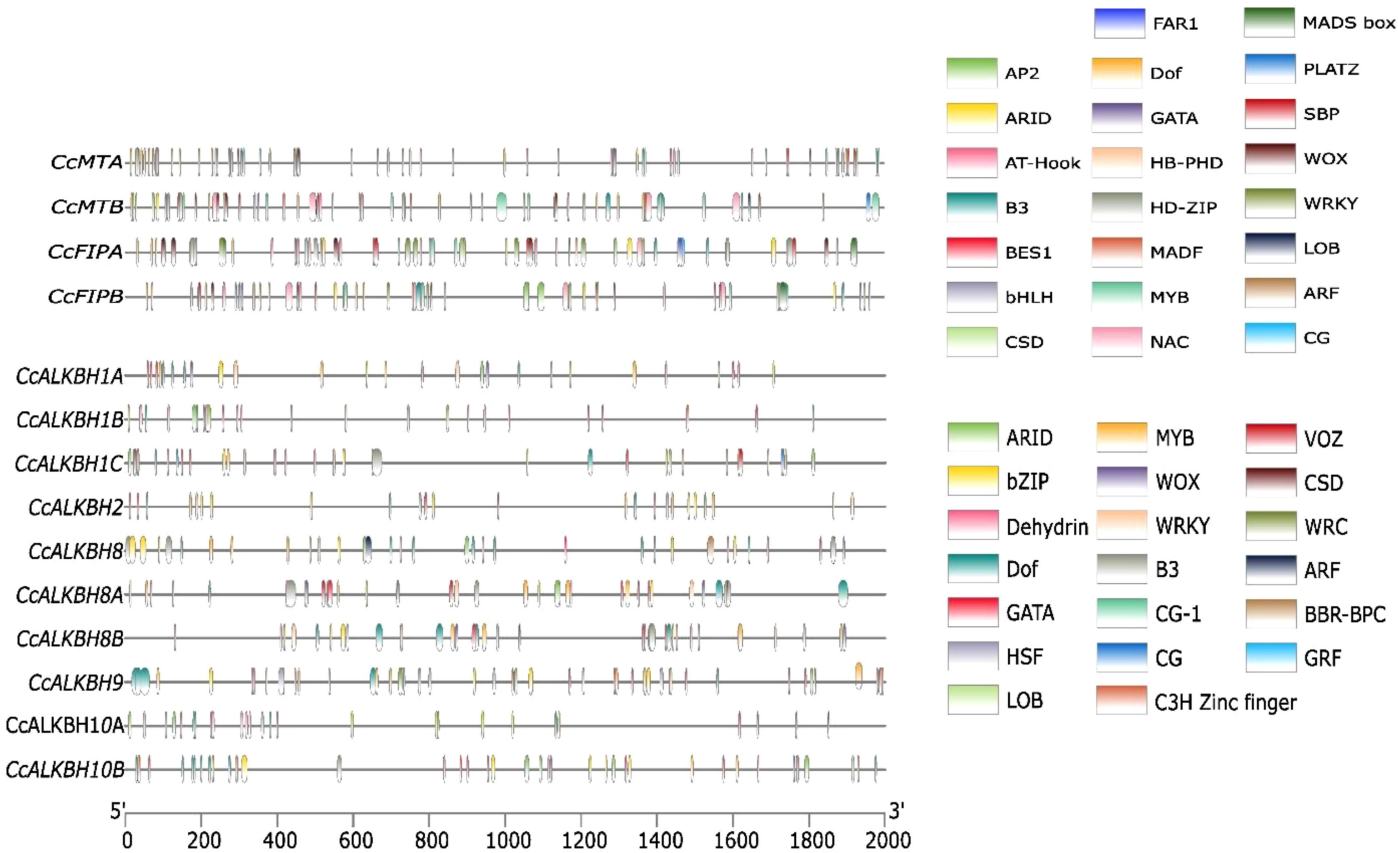
Cis-regulatory elements present in the promoter region of *CcMT*s, *CcFIP*s and *CcALKBH*s genes. Different colour lines represent different cis-regulatory elements.

**Figure 7:**
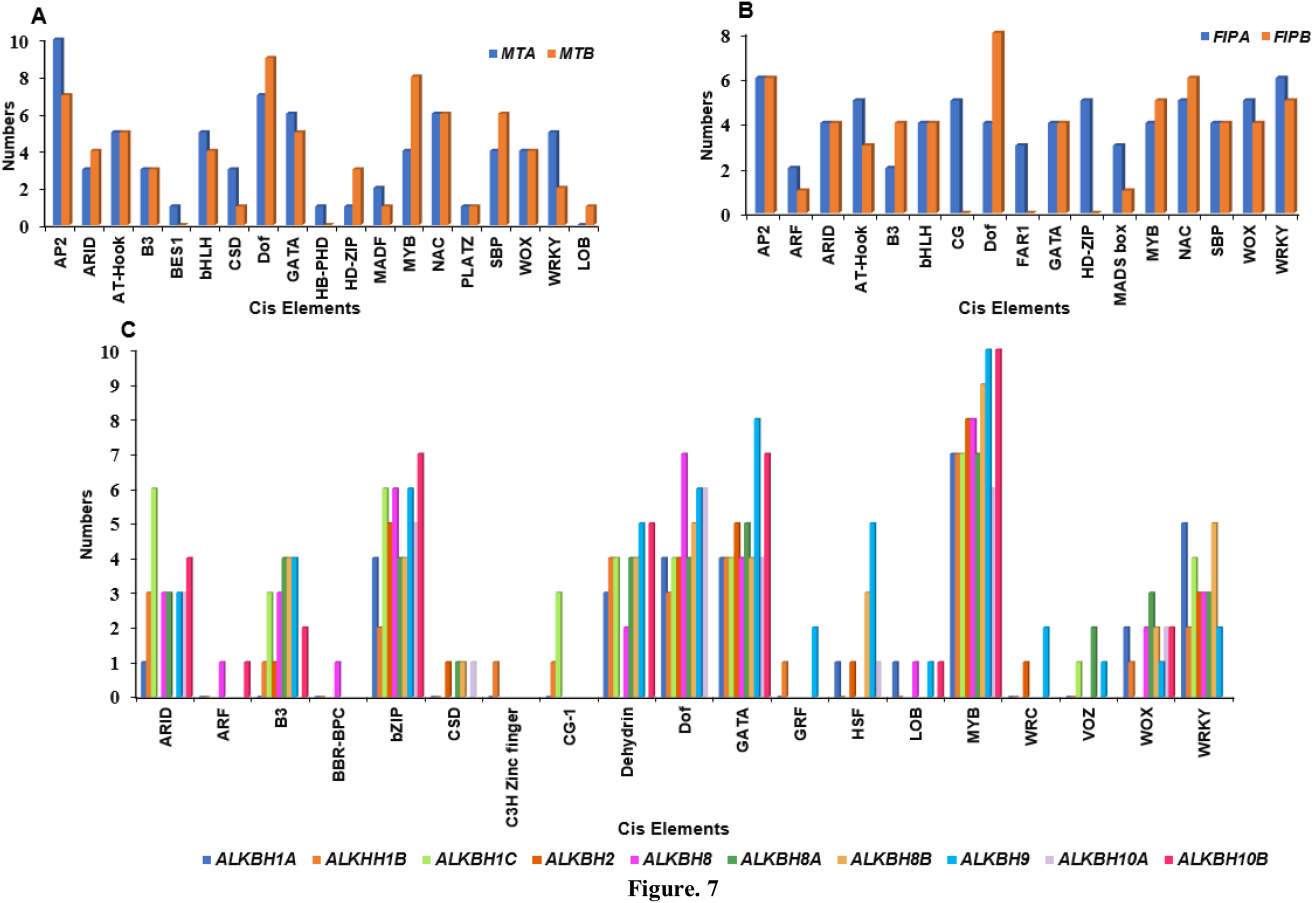
Graph showing enrichment of cis-regulatory elements in identified *MT*s, *FIP*s and *ALKBH*s. **(A)** Enrichment graph for *MTA* (blue bar) and *MTB* (Maroon bar). **(B)** enrichment bar for *FIPA* (blue bar) and *FIPB* (Maroon bar). **(C)** Graph representing *ALKBH*s identifies *cis* elements analysis. Ten different colour bars represent the ten *ALKBH*s genes in pigeon pea.

The cis-regulatory elements in the promoter region of Pigeonpea *MT*s, *FIP*s and *ALKBH*s genes were predicted using the 2kb upstream sequence retrieved from the available database for pigeon pea (LIS database). The identified cis-elements were then selected based on their role in growth and development, hormone response and stress. Growth and development regulatory elements like ARID, AT-Hook, Dof, NAC, LOB, SBP, HD-ZIP, PLATZ and FAR1 were selected. AT-Hook, B3, BBR-BPC, BES1 were selected for hormone response and AP2, bHLH, MADS Box, GATA, WOX, WRKY, C3H-Zinc finger, Dehydrin and VOZ were selected for stress response. Cis-elements varied between genes based on the presence and absence and also on the frequency by which they appear. For instance, both the *MT*s, viz. *MTA* and *MTB*, had almost equal number of cis-elements (**Figure 6**). *MT*s had the highest number of AP2 binding sequences followed by Dof (**Figure 7**). Between the two *FIP*s, *FIPA* had a greater number of elements as compared to *FIPB.* CG, FAR1 and HD-ZIP binding sequences were absent in *FIPB* but present in *FIPA* (**Figure 6**). In case of *ALKBH*s, *CcALKBH9* had the highest number of cis-regulatory elements followed by *CcALKBH8* and *CcALKBH1B* (**Figure 6**). MYB cis-regulatory binding elements were found to be the highest among *ALKBH*s followed by cis-regulatory binding elements for GATA, bZIP and Dof. (**Figure 7**). A table of cis-elements with their numbers for all three genes, viz, *MT*s, *FIP*s and *ALKBH*s, were provided in the **Supplementary Files** (**Supplementary Table 3**).

**Figure 8:**
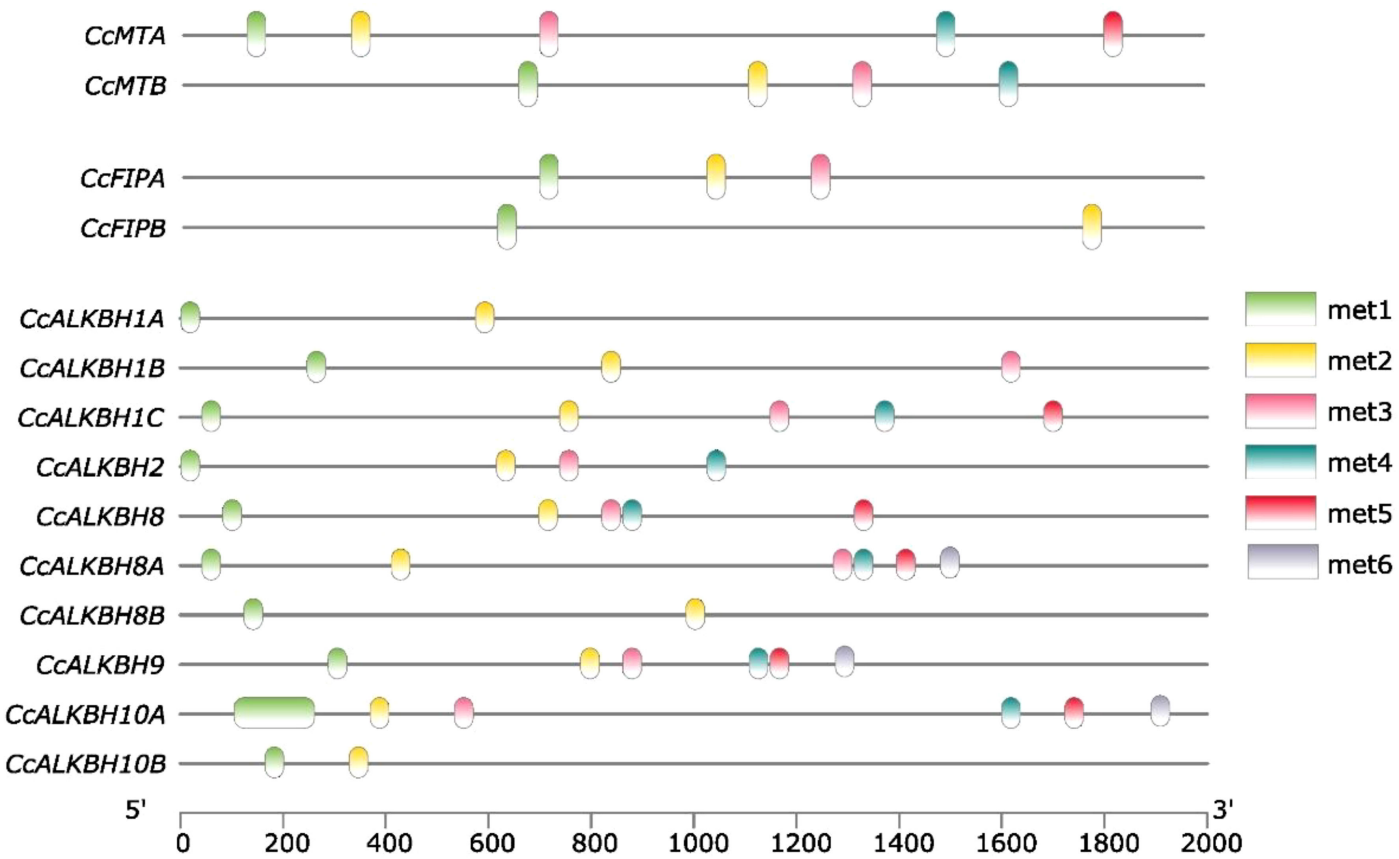
Predicted m6A-methylation position at identified *cis-regulatory* elements in the promoter region of *CcMT*s, *CcFIP*s and *CcALKBH*s genes. Different colour lines indicate the position of methylation.

EpiSemble R-package was used to predict the m^6^A methylation pattern in *MT*s, *FIP*s and *ALKBH*s. This exercise was carried out to understand the epigenetic regulation of the genes. In case of *MT*s five and four methylation sites were found in the upstream 2 kb region (**Figure 8**). For *FIP*s, three and two sites were found for *FIPA* and *FIPB*, respectively (**Figure 8**). Further in case of *ALKBH*s, *CcALKBH9* and *CcALKBH10A* had the highest number of methylation site (six), but the lowest methylation site was found for *CcALKBH1B* and *CcALKBH10B* (two) (**Figure 8**).

### Tissue-specific gene expression analysis

**Figure 9:**
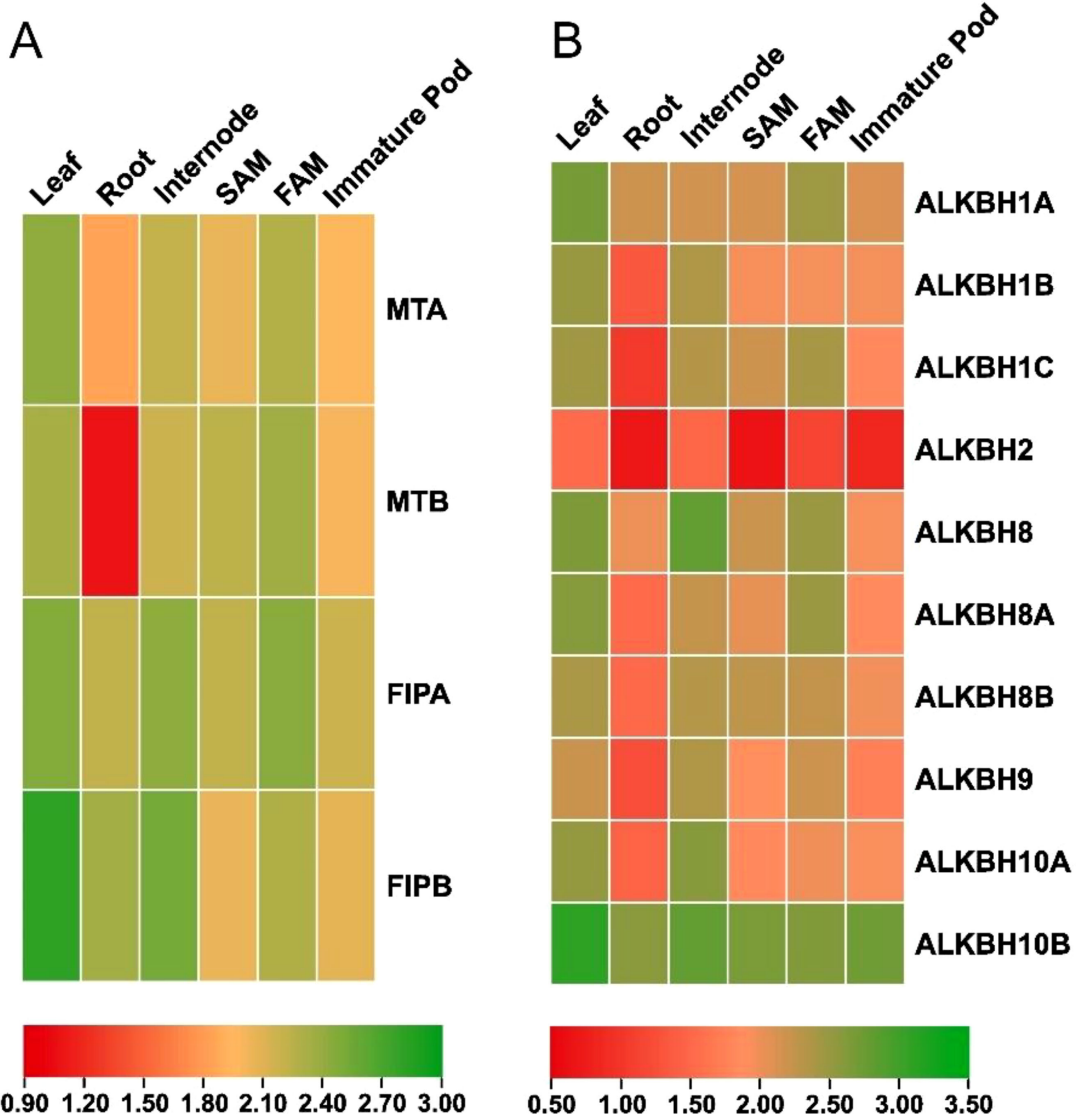
Expression analysis of the identified pigeon pea genes involved in methylation-demethylation. **(A)** Heat map analysis of pigeon pea *MT*s and *FIPs* genes. **(B)** Heat map analysis of pigeon pea *ALKBHs* genes. Column represents different plant tissues and rows represent the identified genes. Significant up-regulationin expression is shown in green, while significant down regulation in expression is shown in red.

Quantitative polymerase chain reaction (q-PCR) was performed to understand the expression pattern of the identified *MT*s, *FIP*s and *ALKBH*s in pigeon pea. Different tissues (leaf, root, internode, shoot apical meristem, flower apical meristem and immature pod) were checked for the relative abundance of the transcripts (**Figure 9**). In case of *MT*s, it was found that overall expression of *CcMTA* was higher in the six selected tissues compared to that of *CcMTB*. The highest expression for *MTA* was observed in leaf tissues (~4.3 fold), while the highest expression for *MTB* was detected in FAM tissues (~3.7 fold). A similar kind of expression pattern was observed for these two genes in other tissues (root, internode, SAM and immature pod), but with varied expression levels i.e., *CcMTA* (~2.5 fold) had significantly higher expression compared to that of *CcMTB* (~1.0 fold) in root tissues. But, *CcMTB* (~3.7 fold) had more expression in SAM tissues compared to that of *CcMTA* (~3.1 fold) (**Figure 9** **).** In case of FIPs, both the genes, viz. *CcFIPA* and *CcFIPB*, showed the highest expression in leaf and internode. However, higher expression of *CcFIPB* was detected in the leaf (~6.0 fold) and root (~4.0 fold), and relatively more expression of *CcFIPA* was detected in FAM (~4.4 fold) tissues (**Figure 9**).

Majority of the genes encoding ALKBHs displayed similar kind of expression patterns with the highest level of expression in leaf tissues, except for *CcALKBH8* (~5.9 fold), *CcALKBH9* (~4.1 fold) and *CcALKBH10A* (~4.9 fold), which showed the highest expression in internode tissue. The second highest expression of seven *ALKBH* genes was also detected in internode tissue, but three genes, viz. *CcALKBH1A* (~4.5 fold), *CcALKBH1C* (~4.3 fold) and *CcALKBH8A* (~4.5 fold) showed the second highest expression in FAM tissue. Among the ten *ALKBH* genes, *CcALKBH10B* had the highest expression in all the six tissues analyzed and *CcALKBH2* had the lowest expression (**Figure 9**). Overall, the highest level of expression of genes encoding MTs, FIPs and ALKBHs was detected in leaf and the lowest expression in root tissues (**Figure 9**).

### Expression profiling of identified genes in biotic and abiotic stress conditions

**Figure 10:**
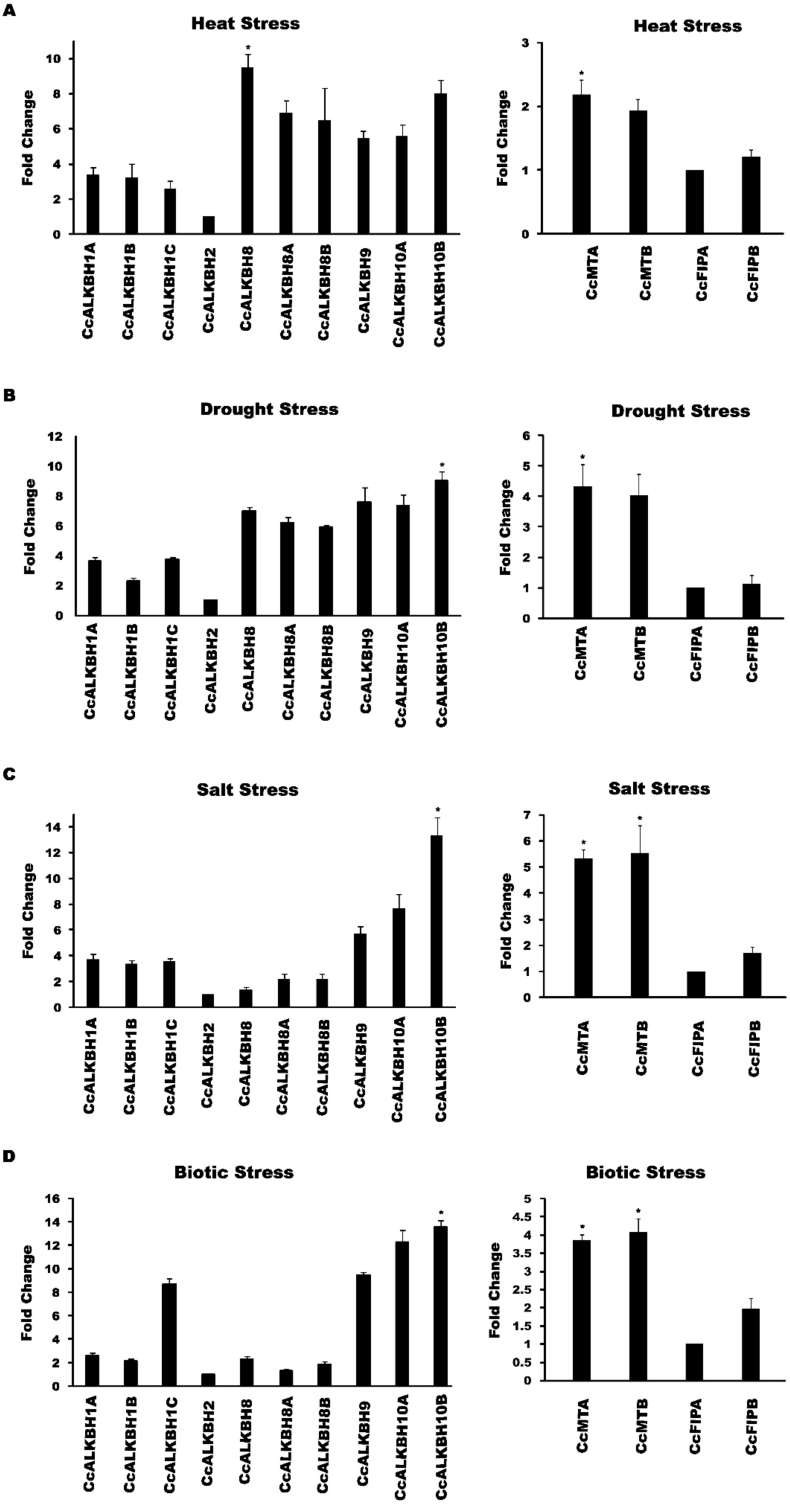
Graphical representations of fold change in expression of *CcMTs, CcFIPs, CcALKBHs* genes of pigeon pea under different stress conditions as revealed by qPCR analysis. **(A)** Heat stress induced change in expression of *CcALKBHs* (demethylase) and *CcMT*s & *CcFIP*s (methyl transferase). **(B)** Expression induction of *CcALKBH*s (demethylases) and *CcMT*s & *CcFIP*s (methyl transferases) during drought stress. **(C)** Salt stress-induced change in expression of *CcALKBH*s (demethylases) and *CcMT*s & *CcFIP*s (methyl transferases). **(D)** Fold change in expression of *CcALKBH*s (demethylases) and *CcMT*s & *CcFIP*s (methyl transferases) upon *H*. *armigera* infestation. Three biological and three technical replicates were taken for expression studies. Star mark indicates the significant difference between the different treatment. For the study of gene expression in abiotic and biotic stress condition three biological and three technical replicates were taken. And mean values were calculated and given error bar (standard error of means). So, Values are the mean ± SE obtained from three independent replicates. At 5% Least significant difference (LSD) was calculated to see the significance of different treatment effect and after that level of significance between and among the treatments in each experiment was checked by performing range test WASP package (AKMU ICAR-CCRI, GOA.).

We wanted to see the expression level changes in the identified genes under various abiotic and biotic stresses. So, we subjected pigeon pea seedlings under various stresses and the morphological changes which was found is provided in **Supplementary Figure 1** Further relative expression of *MT*s, *FIP*s and *ALKBH*s genes of pigeon pea during biotic and abiotic stresses was studied by qPCR analysis. During heat stress the highest induction in expression was observed in *CcALKBH8* (~9.5 fold) followed by *CcALKBH10B* (~8.0 fold), but no induction in expression was found in *CcALKBH2* (~1 fold) compared to that of control (**Figure 10** **).** Among the methyl transferase genes induction in expression was not so prominent, and about two-fold induction in expression was detected for *CcMTA* (~2.1fold) and *CcMTB* (~1.9 fold), whereas very low induction was observed for *CcFIPB* (~1.2 fold) and *CcFIPA* (~1.0 fold) during heat stress (**Figure 10**). Under drought stress, *CcALKBH10B* showed nine-fold more expression, followed by *CcALKBH9* (~7.5 fold) and *CcALKBH10A* (~7.3 fold). *CcALKBH2* (~1.0 fold) showed negligible induction (**Figure 10**). In case of methyl transferase genes, about four-fold induction in expression was observed in *CcMTA* (~4.3 fold) and *CcMTB* (~4.0 fold), but induction was not so prominent in *CcFIPB* (~1.1 fold) and *CcFIPA* (~1.0 fold) (**Figure 10** **).** The highest level of induction in gene expression of 13-fold was detected in *CcALKBH10B* (~13.3 fold) during salt stress. Two other genes, *CcALKBH10A* (~7.6 fold) and *CcALKBH9* (~5.7 fold) showed 8- and 6-fold induction, respectively, during salt stress. Whereas, *CcALKBH2* (~1) showed negligible induction (**Figure 10** **).** Two methyl transferase genes, *CcMTB* (~5.5 fold) and *CcMTA* (~5.3 fold), showed about five-fold more expression during salt stress. Again, *CcFIPB* (~1.6 fold) and *CcFIPA* (~1.0 fold) showed very little induction in expression during salt stress (**Figure 10** **).**

A higher level of induction in gene expression, ranging from 13 to 9-fold, was detected in *ALKBH* genes in pigeon pea upon *H. armigera* infestation. The highest induction was observed in *CcALKBH10B* (~13.5 fold), followed by *CcALKBH10A* (~12.2 fold), *CcALKBH9* (~9.4 fold) and *CcALKBH1C* (~8.6 fold). Again, *CcALKBH2* (~1.0 fold) showed negligible induction during biotic stress (**Figure 10** **).** Less pronounced induction was observed for *MT*s genes with about four-fold induction in *CcMTB* (~4.0 fold) and *CcMTA* (~3.8 fold) followed by 2-fold induction in *CcFIPB* (~1.9 fold). However, induction in *CcFIPA* (~1.0 fold) was not significant (**Figure 10**).

## Discussion

Methylation and demethylation dynamics have a major role in epigenetic regulation of plants growth and development (Huong et al., 2020; Liang et al., 2020) and stress responses (Shoaib et al., 2021). The methylation of adenine (6-methyladenosine, m^6^A) in plants was initially seen in maize, oats, and wheat (Nichols, 1980). mRNAs move to various body parts where they act as potential signaling molecules. The translational state in maize is correlated with m^6^A methylation (Luo et al., 2020). Global m^6^A RNA methylation in seagrass has a significant role in circadian regulation and may have an impact on their photo-biological behavior (Ruocco et al., 2020). Furthermore, m^6^A methylation is required to maintain levels of mature miRNAs and their precursors, as evidenced by a report on its effects on microRNA (miRNA) production in *Arabidopsis* (Bhat et al., 2020). RNA methylation has a role in the mobility and transport of RNA in plants (Yang et al., 2018). Further m^6^A demethylation plays an important role in abiotic stress (heat, drought and salt stress) response (Huong et al., 2020). The methyl transferase (*MT* gene family) and demethylase genes (*ALKBH* gene family) have been identified in the model plant Arabidopsis (Wan et al., 2015) and a major crop plant, rice (Liang et al., 2020). However, the *MTs* and *ALKBHs* gene families are yet to be studied in pigeon pea, an important legume crop. In the present study, we have carried out a genome-wide analysis by comparing the alignments of homologous ALKBH protein sequences from Arabidopsis and pigeon pea to find out methylation and demethylation-related genes. A total of four methylation-related (two methyl transferases, *MT*s and two adaptors proteins for methylation; *FIPA* and *FIPB*) and 10 *ALKBH* (*CcALKBH1A, CcALKBH1B, CcALKBH1C, CcALKBH2, CcALKBH18, CcALKBH8A, CcALKBH8B, CcALKBH9, CcALKBH10, and CcALKBH10B*) family genes had been identified. The identified *MT*s and *ALKBH*s were similar in number as that of tomato and sugar beet genomes, but gene numbers were less than that of Arabidopsis, rice, wheat and Populus. This could be possible because of the evolutionary time gap.

Phylogenetic analysis is used for the identification of orthologous proteins (Bauwens et al., 2018). In the present study, MT and FIP genes were divided into two groups each (*CcMTA*, *CcMTB* and *FIPA*, *FIPB)* and the ALKBH genes were divided into four groups, viz. *CcALKBH1A/1B/1C/2* like, *CcALKBH8/8A/8B* like, *CcALKBH9* like and *CcALKBH10A/10B* like. Whereas in Arabidopsis, one more group was found, i.e., *AtALKBH6* (Mielecki et al., 2012 **),** which was absent in pigeon pea. Among the identified groups, *CcALKBH9* and *CcALKBH10A/10B* are homologs of *AtALKBH9A/9B/9C* and *AtALKBH10A/10B/10C*, respectively, which were reported to carry out m^6^A demethylation (Duan et al., 2017; Martínez-Pérez et al., 2017). Therefore, it is perceived that *CcALKBH9* and *CcALKBH10A/10B* could be putative m^6^A demethylases. However, this needs further validation. The gene structures of *CcMTs, CcFIP and CcALKBHs* were analyzed (**Figure 3B**). It has been found that gene structure for methyl transferase genes is more or less conserved. This conservation of gene architecture for MTs could be to ensure gene stability and integrity and to limit random changes. However, genes encoding FIPs (adaptor protein for methylase transferase) and ALKBHs have shown variation in gene structure. These changes might have occurred during evolution, and this could be the basis for different functions of the identified demethylase genes.

*Cc*MTs had methyl transferase domain, which might be responsible for methylation. For the demethylation activity of ALKBH, one important factor is the presence of the Fe^2+^ binding domain required for its catalytic activity (Fedeles et al., 2015), and all the identified *CcALKBHs* contain the Fe^2+^ binding domain (**Figure 4**). This Fe^2+^ binding domain might mediate the oxidative demethylation of nucleic acids. Additionally, *CcALKBH8* contains a methyl transferase domain, which might be responsible for both methylation and demethylation activity.

Further, *CcALKBH8A* contains an RRM (RNA recognition motif), which is required for tRNA binding and its modification (Pastore et al., 2012). ALKBH of the same sub-group has been found to exhibit a similar pattern in gene structure and conserved motifs, but variation was present among the sub-groups. The conserved motifs analysis of the identified *MT*s, *FIP*s and *ALKBH*s of pigeon pea revealed that a few motifs were conserved across genes but some motifs were unique to some particular genes. The variation in sequence structure and motifs might be responsible for changes in function over a period of time.

Upstream promoter sequences analysis of *MT*s, *FIP*s and *ALKBH*s revealed presence of many regulatory elements related to abiotic stress, hormones and light responses. The promoter sequence of *MT*s harbors more recognition elements for AP2 which has an important role in transcription stimulation in low temperature and water deficit (Sharoni et al., 2011). *MTA* and *MTB* promoter sequences also have presence of GATA and Dof recognition sequence. These elements have role in development and growth of plant (Cai et al., 2020). *FIP* promoter has a high number of recognition elements for Dof which has a role in phytohormone production, seed development and cold stress. Further, *ALKBH* upstream sequence has elements for MYB, which has recently been reported to have a role in m^6^A methylation modification (Xing et al., 2023).

The role of various *MTs* and *ALKBHs* has been characterized in a few plant species. *MTA* and *MTB* are reported to function in embryo development in Arabidopsis (Zhong et al., 2008). *MTA* has also been reported to impart drought tolerance in poplar by regulating the development of trichomes and roots through m^6^A methylation (Lu et al., 2020). FIP37 has been reported to play a role in endosperm and embryo development (Zhong et al., 2008). It was first identified in Arabidopsis as an interacting partner of MTA. Similarly, ALKBHs of Arabidopsis act on different substrates, i.e., *ALKBH2* does repairing of 1-meA and 3-meC, *ALKBH8* takes part in modification of tRNA by hydroxylating mcm^5^U to (S)-mchm^5^U. *AtALKBH6* has been reported to have a role in abiotic stress response where it acts as negative regulator in cold and salt stress but a positive regulator in dehydration stress, viz, heat and drought (Huong et al., 2020). *AtALKBH9B* has been reported to modulate systemic viral infection by demethylating the alfalfa mosaic virus genome (Martínez-Pérez et al., 2017). *AtALKBH10B* has a role in floral transition by affecting the stability of key floral regulators, including FLOWERING LOCUS *T* (*FT*), SQUAMOSA PROMOTER BINDING PROTEIN-LIKE 3 (SPL3) and SPL9 which results in early flowering (Duan et al., 2017). *AtALKBH10B* is also involved in drought tolerance, where it affects m-RNA stability through demethylation of m^6^A (Han et al., 2023). It also modulates ABA response during seed germination (Tang et al., 2021) and was found to impart tolerance to salt stress in Arabidopsis (Shoaib et al., 2021). A recent study showed that in case of cotton, *GhALKBH10B* affects the mRNA stability of genes linked to photosynthesis and GhSnRK2;3, which leads to a negative response to drought stress (Zhang et al., 2024). In case of tomato, *SlALKBH2* has been reported to have RNA demethylase activity, which delays fruit ripening (Zhou et al., 2019).

The qPCR analysis of the identified *MT*s, *FIP*s and *ALKBH*s revealed the changes in the expression level of genes in six different tissues (Leaf, Root, Internode, SAM, FAM and Immature pod). In case of *MT*s, *CcMTA* has a slightly higher expression as compared to *CcMTB*. Similarly, *CcFIPB* showed comparatively higher expression than that of *CcFIPA*. So, *CcMTA* and *CcMTB* could be the probable methyl transferase genes in pigeon pea, and *CcFIPB* might be the adaptor protein that stabilizes the methyl transferase components during methylation process. However, further validation is needed to confirm their function.

*AtALKBH9B* and *AtALKBH10B* have been reported as the major demethylases in Arabidopsis (Duan et al., 2017; Martínez-Pérez et al., 2017). The highest expression of *CcALKBH10B* was detected in different tissues of pigeon pea compared to that of *CcALKBH8*, *CcALKBH10A* and *CcALKBHB9*. Hence, it could be possible that *CcALKBH10B* could be primarily involved in demethylation in pigeon pea as perceived from the expression analysis.

Expression profiling of the *CcMTs*, *CcFIPs* and *CcALKBHs* under abiotic (Heat, Drought and salt) and biotic stress (*H. armigera*) revealed a similar trend of induction in expression. *CcMTA* and *CcMTB* showed similar patterns of induction under both the biotic and abiotic stresses. Similarly, a high level of induction in expression was observed in *CcALKBH8*, CcALKBH*10A* and *CcALKBH10B* under both the biotic and abiotic stress conditions. This indicated that both *CcMTA* and *CcMTB* could be the major methyl transferase genes, and *CcALKBH8*, *CcALKBH10A* and *CcALKBH10B* could be the major demethylase genes in pigeon pea. Arabidopsis demethylase gene*, AtALKBH10B*, was reported to be involved in drought and salt stress tolerance by affecting mRNA stability through demethylation of m^6^A (Shoaib et al., 2021; Han et al., 2023).

## Conclusion

Methylation demethylation dynamics plays an important role in imparting abiotic (like heat, drought and salt stress) and biotic (like against viral infection) tolerance. However, these genes and their function yet to be explored in pigeon pea. Hence, we conducted initial study to find out the different methyltrasferase and demethylase genes present in the pigeon pea genome and their expression pattern in different tissues and stress conditions. Now, from this study the genes which are expressing in response to stress will be selected for functional analysis. Hence this study has its importance by providing the basic knowledge of different methyltransferase and demethylase gene present in pigeon pea and their expression level which will finally help in selection and manipulation of genes for imparting abiotic and biotic stress tolerance.

## Data Availability

The original contributions presented in the study are included in the article/**Supplementary Material**. Further inquiries can be directed to the corresponding author/s.
