# Supplementary material for: Identification of methyltransferase and demethylase genes and their expression profiling under biotic and abiotic stress in pigeon pea (Cajanus cajan [L.] Millspaugh)
Source: Front Plant Sci. 2025 Jan 16;15:1521758. doi: 10.3389/fpls.2024.1521758 (PMC11779730; doi:10.3389/fpls.2024.1521758)
Supplement: Supplementary file 1 [file DataSheet1.docx]

**SUPPLEMENTARY TABLES:**

Table S1: Subcellular localization of identified methylases and demethylases in pigeonpea.

| **Name** | **Function** | **Subcellular localization** |
| --- | --- | --- |
| *CcMTA* | Methylation | Nucleus |
| *CcMTB* | Methylation | Nucleus |
| *CcFIPB* | Methylation/adaptor protein | Nucleus |
| *CcFIPB* | Methylation/adaptor protein | Nucleus |
| *CcALKBH1A* |  | Nucleus |
| *CcALKBH1B* |  | Chloroplast |
| *CcALKBH1C* |  | Nucleus |
| *CcALKBH2* |  | Nucleus |
| *CcALKBH8* |  | Plasma membrane |
| *CcALKBH8A* |  | Nucleus |
| *CcALKBH8B* |  | Nucleus |
| *CcALKBH9* |  | Nucleus |
| *CcALKBH10A* |  | Nucleus |
| *CcALKBH10B* |  | Chloroplast |

Table S2: List of primers used in real-time PCR for expression profiling of *MT*s, *FIP*s and *ALKHB*s genes of pigeonpea

| Oligos name | Sequence (5’ to 3’) | Length (nt) |
| --- | --- | --- |
| ALKB_1A F  ALKB_1A R | GAAGTGGCGATGTTGTTCTT  CGTGTCTCAAGATGGCCTATTT | 20 |
|  |  | 22 |
| ALKB_1B F  ALKB_1B R | GGAAAGCTTGGTCTTCATCAG  CATCCCTCTGATCACCATACA | 21  21 |
| ALKB_1C F  ALKB_1C R | CTTTCCTGATAATTCCCTTCCC  CTTCACTCTCATCCTTGTCTTG | 22  22 |
| ALKB_2 F  ALKB_2 R | CACCTTCATCTGCTGAGAAATC  ACCTAGGTAGTCTCTAGCTTCT | 22  22 |
| ALKB_8 F  ALKB_8 R | CAGAAAGTGAGGAGAGCAGAT  GGTAAATGCCAGGGAACAAAG | 21  21 |
| ALKB_8A F  ALKB_8A R | CTCGATCTTTGCTACTCTTGTC  CTTCTGATGACTCTGCCATTC | 22  21 |
| ALKB_8B F  ALKB_8B R | GGGTTCCTTGATTCTCATGTC  GAGTCAATTGTTGTCCCTTCC | 21  21 |
| ALKB_9 F  ALKB_9 R | ATAAAGACAGGAGAGGAGGTG  TCCGGATCTTGGTGTACTTAG | 21  21 |
| ALKB_10A F  ALKB_10A R | AACCTGTGTCCACTCTTCTT  CCTTCTTCAATGAGAGCATGAG | 20  22 |
| ALKB_10B F  ALKB_10B R | AGTGGCTATGATGAGGCTATG  GTCCCACCTTGAGGAAGTTTA | 21  21 |
| MTA_70 F  MTA_70 R | CGGATTAGTCCCAGAACAAGAA  CCCTCATCAACCAACCTTACA | 22  21 |
| MTB_70 F  MTB_70 R | GGAAGAGGAAGAGGTCAGAAAG   CGGAGTCCAGGTTTCCATATT | 22  21 |
| FIPA F  FIPA R | GTTGAAGAAGCACATGGAAGG  TCTATGGATCTCCCGATTCTTG | 21  22 |
| FIPB F  FIPB R | TTATATCTGCTGGGACCACTC  TGACAATGAATGCAGCTTCC | 21  20 |
| IF4 F (*C. cajan* reference gene) | GCCGAGATCACACAGTCTCA | 20 |
| IF4 R (*C. cajan* reference gene) | ACCACGAGCCAAAAGATCAG | 20 |

Table S3: Numbers of identified *cis* elements in *MT*s, *ALKHB*s and *FIP*s genes of pigeonpea

| Gene | Cis regulatory elements | Frequency of Elements |
| --- | --- | --- |
| *MTA* | AP2  ARID  AT-Hook  B3  BES1  bHLH  CSD  Dof  GATA  HB-PHD  HD-ZIP  MADF  MYB  NAC  PLATZ  SBP  WOX  WRKY | 10  03  05  03  01  05  03  07  06  01  01  02  04  06  01  04  04  05 |
| TOTAL | 18 |  |
| *MTB* | AP2  ARID  AT-Hook  B3  bHLH  CSD  Dof  GATA  HD-ZIP  LOB  MADF  MYB  NAC  PLATZ  SBP  WOX  WRKY | 07  04  05  03  04  01  09  05  03  01  01  08  06  01  06  04  02 |
| TOTAL | 17 |  |
| *FIPA* | AP2  ARF  ARID  AT-Hook  B3  bHLH  CG  Dof  FAR1  GATA  HD-ZIP  MADS box  MYB  NAC  SBP  WOX  WRKY | 06  02  04  05  02  04  05  04  03  04  05  03  04  05  04  05  06 |
| TOTAL | 17 |  |
| *FIPB* | AP2  ARF  ARID  AT-Hook  B3  bHLH  Dof  GATA  MADS box  MYB  NAC  SBP  WOX  WRKY | 06  01  04  03  04  04  08  04  01  05  06  06  04  05 |
| TOTAL | 14 |  |
| *ALKHB1A* | ARID  bZIP  Dehydrin  Dof  GATA  HSF  LOB  MYB  WOX  WRKY | 01  04  03  04  04  01  01  07  02  05 |
| TOTAL | 10 |  |
| *ALKHH1B* | ARID  B3  bZIP  C3H Zinc finger  CG-1  Dehydrin  Dof  GATA  HSF  Myb  WOX  WRKY | 03  01  02  01  01  04  03  04  01  07  01  02 |
| TOTAL | 12 |  |
| *ALKHB1C* | ARID  B3  bZIP  CG  Dehydrin  Dof  GATA  MYB  VOZ  WRKY | 06  03  06  03  04  04  04  07  01  04 |
| TOTAL | 10 |  |
| *ALKHB2* | B3  bZIP  CSD  Dof  GATA  HSF  MYB  WRC  WRKY | 01  05  01  04  05  01  08  01  03 |
| TOTAL | 9 |  |
| *ALKHB8* | ARF  ARID  B3  BBR-BPC  bZIP  Dehydrin  Dof  GATA  LOB  MYB  WOX  WRKY | 01  03  03  01  06  02  07  04  01  08  02  03 |
| TOTAL | 12 |  |
| *ALKHB8A* | ARID  B3  bZIP  CSD  Dehydrin  Dof  GATA  MYB  VOZ  WOX  WRKY | 03  04  04  01  04  04  05  07  02  03  03 |
| TOTAL | 11 |  |
| *ALKHB8B* | B3  bZIP  CSD  Dehydrin  Dof  GATA  HSF  MYB  WOX  WRKY | 04  04  01  04  05  04  03  09  02  05 |
| TOTAL | 10 |  |
| *ALKHB9* | ARID  B3  bZIP  Dehydrin  Dof  GATA  GRF  HSF  LOB  MYB  VOZ  WOX  WRC  WRKY | 03  04  06  05  06  08  02  05  01  10  01  01  01  02 |
| TOTAL | 14 |  |
| *ALKHB10A* | ARID  Dof  CSD  bZIP  GATA  WOX  HSF  Myb | 03  06  01  05  04  02  01  06 |
| TOTAL | 08 |  |
| *ALKHB10B* | ARF  ARID  B3  bZIP  Dehydrin  Dof  GATA  WOX  LOB  MYB | 01  04  02  07  05  08  07  02  01  10 |
| TOTAL | 10 |  |


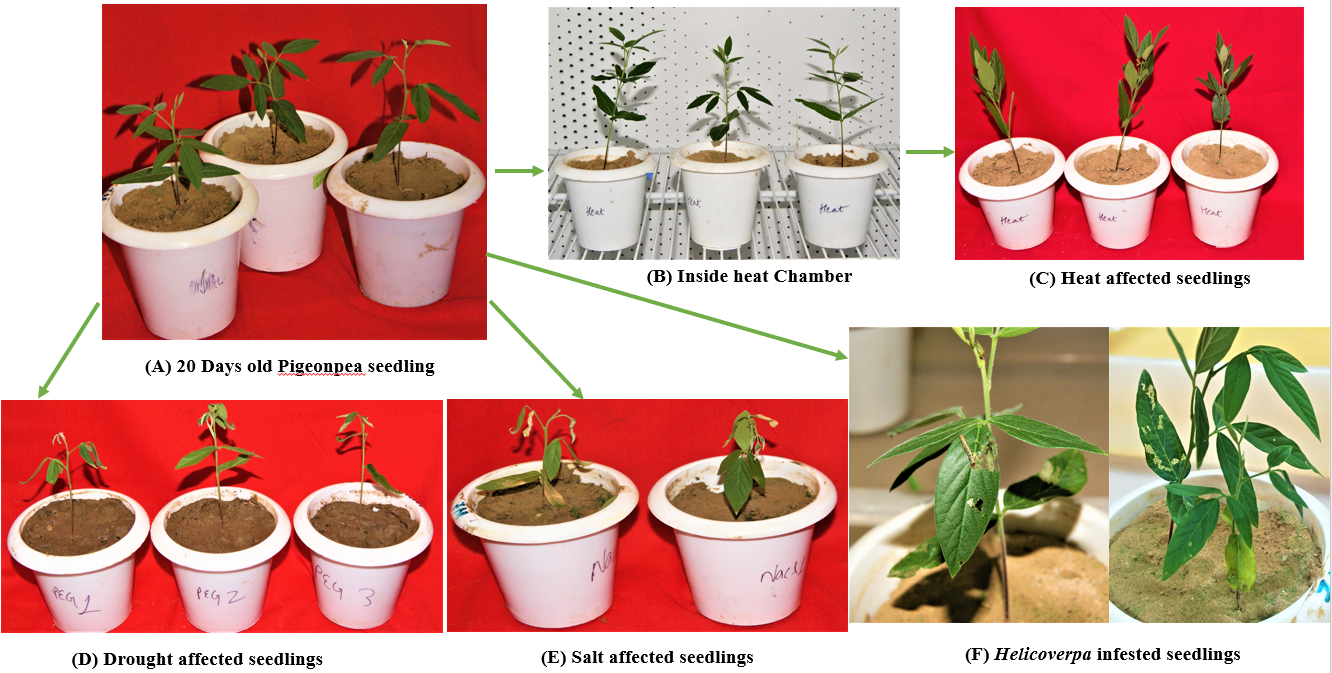


Supplementary Fig.1: Pigeonpea plants subjected to biotic and abiotic stress conditions. (A) Twenty days old normal pigeonpea seedlings (control). (B) Seedlings kept under heat stress (42 ^o^C & 60% RH) (C) Heat stressed pigeonpea seedlings. (D) Drought stressed (20% PEG; 200 ml poured to each seedling) pigeonpea seedlings. (E) Salt stressed pigeonpea seedlings (150 mM NaCl; 200ml poured to each seedling) seedlings. (F) *Helicoverpa armigera* infested (two second instar larvae released on each seedling) pigeonpea seedlings.
